# Interprofessional Education: An Innovative Approach to Increase the Human Immunodeficiency Virus Workforce

**DOI:** 10.1093/ofid/ofad560

**Published:** 2023-11-07

**Authors:** Anna K Person, Sadie J Harris, Jennifer Burdge, Amy V Blue, Nicole E Leedy, Evelyn Villacorta, Julie Ann Justo, Erik Black, Jennifer Janelle, Divya Ahuja, Cody A Chastain

**Affiliations:** Southeast AIDS Education and Training Center, Division of Infectious Diseases, Department of Medicine, Vanderbilt University Medical Center, Nashville, Tennessee, USA; Southeast AIDS Education and Training Center, Division of Infectious Diseases, Department of Medicine, Vanderbilt University Medical Center, Nashville, Tennessee, USA; Southeast AIDS Education and Training Center, Division of Infectious Diseases, Department of Medicine, Vanderbilt University Medical Center, Nashville, Tennessee, USA; Office of Interprofessional Education and College of Public Health and Health Professions, University of Florida, Gainesville, Florida, USA; Division of Infectious Diseases, Department of Internal Medicine, University of Kentucky College of Medicine, Lexington, Kentucky, USA; Division of Infectious Diseases, Department of Internal Medicine, University of Kentucky College of Medicine, Lexington, Kentucky, USA; College of Pharmacy, University of South Carolina,Columbia, South Carolina, USA; Office of Interprofessional Education, University of Florida Health Science Center, University of Florida, Gainesville, Florida, USA; Division of Infectious Diseases and Global Medicine, Department of Medicine, University of Florida, Gainesville, Florida, USA; Division of Infectious Diseases, Department of Internal Medicine, University of South Carolina, Columbia, South Carolina, USA; Southeast AIDS Education and Training Center, Division of Infectious Diseases, Department of Medicine, Vanderbilt University Medical Center, Nashville, Tennessee, USA

**Keywords:** interprofessional education, medical education, people with HIV, Southeast AIDS Education and Training Center, workforce shortage

## Abstract

Ending the human immunodeficiency virus (HIV) epidemic relies on a robust clinical workforce. The Southeast AIDS Education and Training Center's interprofessional education program is a novel approach to increasing the interest and ability of early health professional learners to provide high-quality, comprehensive, person-first care for people with HIV.

**Key Points:** Interprofessional education (IPE) focusing on multidisciplinary care for people with HIV can serve as a novel way to increase the HIV workforce. This brief report describes the IPE program of the Southeast AIDS Education and Training Center.

## Teaser

Interprofessional education focusing on multi-disciplinary care for people with HIV can serve as a novel way to increase the HIV workforce. This brief report describes the IPE program of the Southeast AIDS Education and Training Center.

There is longstanding concern that the human immunodeficiency virus (HIV) clinician workforce is insufficient to meet the increasing demand for HIV-related services [1, 2]. In the United States (US), the South has the greatest burden of new HIV diagnoses with patients experiencing the lowest rates of viral suppression; yet an astounding 80% of counties in this region lack experienced HIV providers [3, 4]. For some time, the supply of clinicians entering the workforce has been outnumbered by the demand for HIV healthcare services [5].

The AIDS Education and Training Center (AETC) program was established by the Health Resources and Services Administration (HRSA) with a goal of strengthening the HIV workforce and improving outcomes along the HIV care continuum [6, 7]. Initially, the efforts of AETCs were directed at providers already in the workforce. Over time, it became clear that the HIV workforce shortage is also due to lack of comfort, knowledge, and skill in providing HIV prevention and care among health professional students entering the workforce; thus, the AETC's target populations were expanded.

In 2015, HRSA included a requirement for regional AETCs to partner with accredited programs in medicine, nursing, dentistry, pharmacy, public health, allied health, and/or behavioral health to prepare future healthcare professionals to provide care for people with HIV (PWH) through collaborative practice, noting that interprofessional education (IPE) has been utilized in managing the care of PWH [8, 9]. In response, the Southeast (SE) AETC executed an IPE program with 4 regional partners: University of Florida, University of Kentucky, University of South Carolina, and Vanderbilt University Medical Center. This article aims to illustrate the positive effect of these IPE programs on the HIV workforce.

## SE AETC IPE PROGRAM

The SE AETC developed a vision for IPE guided by the 4 broad Interprofessional Education Collaborative (IPEC) Competencies, which consists of 4 operational guidelines and 9 learning objectives (available in the Supplementary Materials). The specific operational guidelines of the SE AETC IPE program is to (1) cultivate respectful professionals, (2) prepare a collaborative practice–ready workforce, (3) improve health delivery systems, and (4) create self-directed lifelong learners. HIV-specific context is given to these competencies through 9 learning objectives. These learning objectives describe outcomes but are intentionally nonspecific in how they are achieved. This is important given the institutional diversity within the region, which includes varying educational degree programs, clinical infrastructure, and clinical demographics. Each site accepts as few as 8 and as many as 800 students annually, and the length of each program ranges from 2 weeks to 2 years. Educational modalities include didactic sessions, small group work, collaborative activities, case studies, seminars, patient simulations, and preceptor-led clinical work. Each program is required to include 4 health professions (medicine, nursing, social work, and pharmacy students, with 1 of those professions specifically focused on mental health), but they may additionally include dental, public health, counseling, physical therapy, speech-language pathology, or other professional groups. Standardized core program requirements can be completed on a timeframe that best fits each site and includes 20 hours of IPE coursework (at least 8 of those being HIV-related), a capstone project, and at least 40 hours of clinical team–based care in an HIV or LGBTQ (lesbian, gay, bisexual, transgender, and queer)–focused clinic with patient interaction. All IPE programs consist of a “modular” design (ie, curriculum designed to be flexible within a complex health profession educational program).

## METHODS

Given the HIV workforce shortage facing the US, the authors aimed to explore the effects of the IPE program on comfort levels in caring for PWH as well as interest in HIV as a potential career focus. Standardized surveys (available in the Supplementary Materials) were administered at the University of Kentucky, the University of South Carolina, and Vanderbilt University Medical Center. These tools were given to the students at the beginning of their program and at the end of their program to assess participants’ pre-IPE experience (n = 177) and post-IPE experience (n = 136) regarding comfort level and ability to treat PWH. The IPE program at the University of Florida focused on learners earlier in their health professional training; as such, all data elements were not collected for this learner population since many survey items did not apply (n = 6102). To analyze comments about the IPE experience, in vivo coding was utilized to identify emergent themes. All data collected were for educational quality improvement and program evaluation; as such, institutional review board approval was not pursued.

### Quantitative Outcomes

From 2015 to 2022, SE AETC IPE student participation included >6000 students. The students at the University of Kentucky, the University of South Carolina, and Vanderbilt University Medical Center were asked to rate their comfort level and ability to perform professional care to PWH before and after their IPE experience. Table 1 illustrates how students reported an increase in both their comfort level of providing professional care for PWH (increasing from an average of “neither comfortable nor uncomfortable” to an average of “very comfortable”) and in their ability to provide HIV-related services upon completion of their IPE experience (increasing from an average of “needs improvement” to an average of “very good”).

**Table 1.** Southeast AIDS Education and Training Center’s Interprofessional Education Program Outcomes: Comfort and Self-assessed Ability to Perform HIV-Related Services

Outcome	IPE Experience			
	Baseline (n = 177)		Follow-up (n = 136)	
	Median (IQR)	No.	Median (IQR)	No.
Comfort level of providing professional care for PWH^a^	3 (2–4)	48	5 (4–5)	46
*Ability to perform HIV-related services* ^b^				
HIV education and counseling	2 (1–3)	173	4 (3–4)	128
PrEP assessment and prescribing	2 (1–2)	162	4 (3–4)	118
HIV testing	2 (1–3)	159	4 (3–5)	112
Interpretation of HIV testing results	2 (1–3)	162	4 (3.25–5)	122
Linkage to HIV care	2 (1–3)	173	4 (3.25–5)	122
Engagement and retention	2 (1–3)	170	4 (3–4)	124
Prescribing, managing, and monitoring ART	2 (1–2)	153	4 (3–4)	107
ART adherence	2 (1–3)	167	4 (3–5)	121
Hepatitis B and/or C coinfection	2 (2–3)	163	3 (3–4)	117
Mental health disorders	3 (2–3)	171	4 (3–4)	127
Substance use disorders	3 (2–3)	171	4 (3–4)	126
Other chronic medical conditions	3 (2–3)	170	4 (3–4)	123
Sexually transmitted infections	2.5 (2–3)	168	4 (3–4)	124
Opportunistic infections	2 (2–3)	167	4 (3–4)	120
Delivering team-based, interdisciplinary care	2 (2–3)	171	4 (4–5)	130
Providing services to culturally diverse PWH	2 (2–3)	171	4 (4–5)	128
Care coordination for nonmedical needs	2 (2–3)	170	4 (3–5)	127

Data are from 2018 to the present (as available), from Kentucky, South Carolina, and Tennessee.

Abbreviations: ART, antiretroviral therapy; HIV, human immunodeficiency virus; IQR, interquartile range; IPE, interprofessional education; PrEP, preexposure prophylaxis; PWH, people with human immunodeficiency virus.

^a^Scale: 1 = not comfortable, 2 = somewhat uncomfortable, 3 = neither comfortable nor uncomfortable, 4 = somewhat comfortable, 5 = very comfortable.

^b^Scale: 1 = needs considerable improvement, 2 = needs improvement, 3 = adequate, 4 = very good, 5 = excellent.

In addition to increasing overall comfort levels caring for PWH and abilities to perform many HIV-related services, many students from the IPE program have also sought a career in the HIV workforce after completion of the program. Since the initiation of the SE AETC IPE program in 2015, at least 15 learners in Kentucky, South Carolina, and Tennessee transitioned to professional roles serving PWH (including social workers, pharmacists, physician assistants, and psychiatric nurse practitioners). Additionally, most IPE students since 2015 reported an interest to engage in HIV care in some capacity in their future careers, with 73% stating they expect to provide services directly to people with HIV, 22% reporting they are unsure, and only 5% reporting that they do not expect to provide these services.

### Qualitative Outcomes

**Figure 1.**
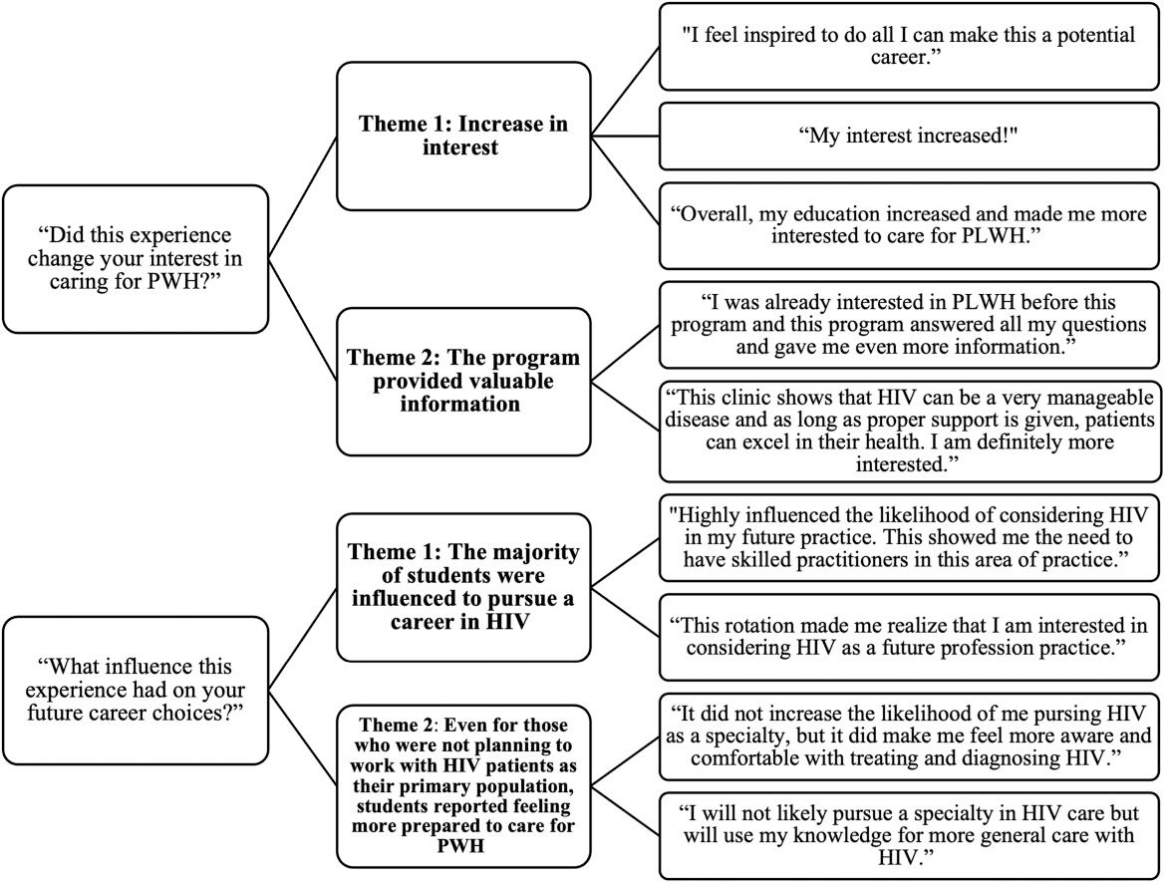
Qualitative emergent themes and associated learner quotes. Abbreviations: HIV, human immunodeficiency virus; PLWH, people living with human immunodeficiency virus; PWH, people with human immunodeficiency virus.

Figure 1 illustrates the qualitative themes from students over 4 years (ie, 2018–2019, 2019–2020, 2020–2021, and 2021–2022). After completing an SE AETC IPE program, students were asked if this experience changed their interest in caring for PWH. Two themes emerged: (1) Interest in HIV care increased following the IPE experience; and (2) the IPE program provided valuable information about HIV. Similarly, after completing their program, students were asked what influence this experience had on their future career choices. Two additional themes emerged: (1) Most of the students were influenced to pursue some sort of career in HIV medicine; and (2) even for those *not* planning to work with PWH as their primary population, students reported feeling more prepared to care for PWH.

## DISCUSSION

There is a crisis in the field of HIV care, with an aging workforce, fewer individuals choosing to specialize in the field of HIV medicine, and many providers being called to serve the needs of other pandemics (eg, coronavirus disease 2019) [1–5]. The current supply of providers is not predicted to meet increasing demand for HIV services [5], and any intervention to increase HIV workforce strength should be explored. Traditionally, the route to HIV medicine is through an infectious diseases (ID) fellowship. However, ID programs frequently do not fill to capacity [10], and graduating ID fellows alone are insufficient to fill the HIV workforce gap. A well-rounded, diverse workforce is needed to provide important the wraparound services PWH need—one that includes family medicine physicians, internal medicine physicians, physician assistants, nurse practitioners, counselors, social workers, pharmacists, dentists, nurses, and others. Existing pathways for healthcare providers to enter the HIV workforce include HIV-focused fellowships; the American Academy of HIV Medicine certification program for physicians, nurse practitioners, physician assistants, and pharmacists; ID/HIV-focused pharmacy residencies; and the Association of Nurses in AIDS Care. However, these rely on a preexisting interest in this area of healthcare, which needs to be fostered early in training. The SE AETC IPE program focuses on early learners who have yet to finalize their career pathways, with a focus on encouraging holistic, expert, person-first care of PWH. Even if learners do not pursue solely an HIV-focused career, being familiar with HIV prevention and treatment, best practices for sexual health history taking, screening and treatment, and supportive and empathetic care for PWH and those at risk for HIV can significantly contribute to ending the HIV epidemic. The authors have shown through descriptive analysis as well as quantitative and qualitative data that IPE programs may support HIV workforce development across health professional training programs.

Limitations include the lack of generalizability across all health professional education settings; incomplete survey responses of IPE participants; and the potential for other factors impacting participant interest in HIV care.

## CONCLUSIONS

AETCs have been valuable in supporting and expanding the HIV workforce in the US. The addition of IPE programs offers a novel way to grow the HIV workforce across multiple health professions simultaneously. The authors report on the success of the SE AETC IPE program in addressing the Southeast US region's HIV workforce. Quantitative and qualitative data show that after participating in the IPE program, students are more comfortable and interested in caring for PWH. The authors believe IPE programs to be an effective and complementary tool to further augment efforts to grow the HIV workforce.

## Supplementary Material

ofad560_Supplementary_Data

## Supplementary Data

Supplementary materials are available at *Open Forum Infectious Diseases* online. Consisting of data provided by the authors to benefit the reader, the posted materials are not copyedited and are the sole responsibility of the authors, so questions or comments should be addressed to the corresponding author.

## Notes

***Acknowledgments.*** The authors thank all people with HIV who participate in the SE AETC IPE program and provide valuable opportunities for learning. Additionally, the authors thank the faculty, clinicians, and staff who support IPE program with their expertise and passion to end the HIV epidemic. Finally, the authors would like to thank the IPE students who strive to be lifelong learners in HIV healthcare and valued HIV workforce members.

***Author contributions.*** A. K. P. conceived the manuscript. All authors contributed to the acquisition of data, providing content, and editing. E. V. and S. J. H. analyzed and prepared the data. A. K. P., C. A. C., and S. J. H. performed the final composition. All authors reviewed and approved the final report.

***Patient consent.*** This manuscript does not include factors necessitating patient consent. The design of the work conforms to standards currently applied in the country of origin and the AETC.

***Financial support.*** This program (the AETC and SE AETC's IPE program) and all authors of this report are supported by the Health Resources and Services Administration (HRSA) of the US Department of Health and Human Services (HHS) under grant number U1OHA30535 as part of an award totaling $4.2 million. ​
