# Supplementary material for: Interprofessional Education: An Innovative Approach to Increase the Human Immunodeficiency Virus Workforce
Source: Open Forum Infect Dis. 2023 Nov 7;10(11):ofad560. doi: 10.1093/ofid/ofad560 (PMC10665034; doi:10.1093/ofid/ofad560)
Supplement: ofad560_Supplementary_Data [file ofad560_supplementary_data.zip › Supplementary Material 3 (IPE Student Assessment Follow-Up.pdf]

Interprofessional Education Project Student Assessment (IPE-SA) One-Time Follow-Up

Instructions: This assessment is to be completed by students who receive HIV IPE training at each IPE Project participating health professional program. Students may be pre-license students or post-license practitioners. The *IPE-SA One-Time Follow-Up* is to be administered immediately after the end of HIV IPE training or program completion, or within two weeks.

Please note, only individuals approved by or directly involved in the AETC Evaluation will use the information collected by this survey.

BACKGROUND INFORMATION

1. **Please provide your email address (or participant ID).**
   The AETC Program uses a participant ID to track participation in training events. Please use the same ID for all your AETC events/trainings.

   ____

2. **What is your discipline/area of study?** *(Select one)*

   [ ] Advanced Practice Nurse/Nurse Practitioner
   [ ] Medicine
   [ ] Dentistry
   [ ] Mental/ behavioral health
   [ ] Nursing
   [ ] Pharmacy
   [ ] Physician Assistant
   [ ] Social work
   [ ] Public Health
   [ ] Dietetics or Nutrition
   [ ] Health Administration
   [ ] Optometry
   [ ] Other health professional program, specify: ______________________________

3. **What is the name of your academic institution and specific health profession program?**
   (e.g., AETC University – School of Medicine)

   Institution: ____

   Specific health professional program: ____

4. **Please answer the following questions about your academic program and status in the program:**

4a. How long is your program or course of study?
(i.e., if 4 years, enter '4' for years; if 1.5 years, then enter '1' for year and '6' for months)

_____*(years)* _____*(months)*

4b. What program year are you currently enrolled in?
If you are a part-time student, indicate the "year of study" you are currently in in your program, rather than the actual number of years you have been in the program.

[ ] 1st year
[ ] 2nd year
[ ] 3rd year
[ ] 4th year
[ ] 5+ years
[ ] Program completed/graduated

4c. Please select the category that best describes your current licensure status:

[ ] Pre-license student
[ ] Post-license practitioner
[ ] Other, please specify: ____

INTERPROFESSIONAL EDUCATION AND INTERPROFESSIONAL HEALTH CARE TEAM FUNCTIONING

The next set of questions ask about your knowledge and attitudes related to interprofessional care and practice to improve HIV care outcomes. Interprofessional education promotes collaborative and integrated learning among two or more types of health professionals (pre-license students and/or post-license practitioners) from different disciplines in order to encourage safe, high quality, accessible, patient-centered care and ultimately, improve health outcomes.

5. **Rate your <u>current level of knowledge</u> on the ideal functioning of interprofessional health care teams.**

[ ] Needs considerable improvement
[ ] Needs improvement
[ ] Adequate
[ ] Very good
[ ] Excellent

6. **Select the response category that best reflects the degree to which you agree or disagree with the following statements regarding interprofessional education.**

	Strongly Disagree	Disagree	Neither	Agree	Strongly Agree
Learning with students from other professions is helpful toward becoming a more effective health care professional	○	○	○	○	○
Working in an interprofessional manner complicates the delivery of care	○	○	○	○	○
To be effective, team members should understand the roles and responsibilities of their fellow interprofessional team members	○	○	○	○	○

The next set of questions are about your current ability to work as a member of an interprofessional health care team, either through a student practicum, preceptorship, residency, patient simulation exercises, or other hands-on clinical training experience where you may have had the opportunity to work with individuals from different health care disciplines to provide patient care.

7. **Please rate your current ability to perform the following functions as part of an interprofessional health care team.**

	Needs considerable improvement	Needs improvement	Adequate	Very good	Excellent
Develop trusting relationships with patients/clients and their families	○	○	○	○	○
Involve patients/clients in decision-making	○	○	○	○	○
Provide constructive feedback to team members on their performance	○	○	○	○	○
Respond to feedback from team members on your performance	○	○	○	○	○
Express opinions in a group, even when others disagree	○	○	○	○	○
Justify recommendations/actions in-person, with more senior people	○	○	○	○	○
Address conflict and differences of opinions among interprofessional team members	○	○	○	○	○
Develop an interprofessional patient/client care plan	○	○	○	○	○

8. **Please select the opportunities you have had to participate on an interprofessional health care team, since starting your training at this school/university/health professional program.**
(Select all that apply)

[ ] Clinical practicum
[ ] Clinical preceptorship
[ ] Clinical rotation
[ ] Internship, residency, or fellowship
[ ] Other hands-on clinical training experience, please specify: ____________________________
[ ] I have not had any opportunities to participate as part of an interprofessional health care team

HIV-RELATED CARE AND SERVICES PROVIDED BY AN INTERPROFESSIONAL TEAM

The next set of questions are about your current ability related to providing HIV-related care and services. When responding, consider the training and education you have received on these topics. Think about your ability to perform these tasks in any hands-on learning opportunities you may have had providing care to clients/patients (e.g., practicum, preceptorship, residency, or other hands-on training experience, patient simulations, etc.).

9. Please rate your current ability to perform HIV-related services listed below in the context of an interprofessional care team.
Please select "N/A" if the service does not apply to your discipline or area of study.

	Needs considerable improvement	Needs improvement	Adequate	Very good	Excellent	N/A
**HIV Prevention**						
HIV education and counseling	○	○	○	○	○	○
PrEP assessment and prescribing	○	○	○	○	○	○
**HIV Testing**						
HIV testing	○	○	○	○	○	○
Interpretation of HIV testing results	○	○	○	○	○	○
**HIV Care and Treatment**						
Linkage to HIV care	○	○	○	○	○	○
Engagement and retention	○	○	○	○	○	○
Prescribing, managing, and monitoring antiretroviral therapy	○	○	○	○	○	○
Antiretroviral therapy adherence	○	○	○	○	○	○
**Screening, Evaluation, and Management of Co-Occurring Conditions**						
Hepatitis B and/or C co-infection	○	○	○	○	○	○
Mental health disorders	○	○	○	○	○	○
Substance use disorders	○	○	○	○	○	○
Other chronic medical conditions	○	○	○	○	○	○
Sexually transmitted infections	○	○	○	○	○	○
Opportunistic infections	○	○	○	○	○	○
**HIV Service Delivery**						
Delivering team-based, interdisciplinary care	○	○	○	○	○	○
Providing services to culturally diverse people with HIV	○	○	○	○	○	○
Care-coordination for non-medical needs	○	○	○	○	○	○
**Other HIV-Related Service**						
Other, please specify: ______________________	○	○	○	○	○	○

HIV IPE TRAINING RECEIVED & POST-TRAINING PLANS

The next set of questions asks about the HIV IPE training you received at your health professional program and your plans for providing care and services to clients/patients with HIV in your current or future work.

10. Thinking back about the HIV IPE training you received at your health professional program, to what extent do you intend to apply the knowledge and skills you learned to your future work?

[ ] Not at all
[ ] A little
[ ] A moderate amount
[ ] Quite a bit
[ ] A great deal

11. Please select one statement that best describes your employment status. *(Select one)*

[ ] I am currently employed/working *(Skip to Question 12)*
[ ] I am hired and will be working *(Skip to Question 12)*
[ ] I will seek employment after completing my program *(Continue to Question 11a)*
[ ] I will continue professional development or study,
please specify: ____ *(Continue to Question 11a)*
[ ] Other, please specify: ____ *(Continue to Question 11a)*

Thinking about your future work, please answer the following questions:

11a. In your future work setting, do you expect to have direct interactions with clients/patients?

[ ] Yes *(Continue to Question 11b)*
[ ] No *(Skip to Question 11c)*
[ ] Not sure *(Skip to Question 11c)*

11b. In your role at your future work setting….

	Yes	No	Not sure
Do you expect to provide HIV prevention and testing services to clients/patients?	○	○	○
Do you expect to assess for and prescribe HIV PrEP to clients/patients?	○	○	○
Do you expect to provide services directly to people with HIV?	○	○	○

11c. **In your future work setting, what will be your <u>primary</u> profession/occupation?**
(Select one)

[ ] Dentist
[ ] Other Dental Professional
[ ] Nurse Practitioner or other Nursing Professional who prescribes
[ ] Nurse Professional who does not prescribe
[ ] Midwife
[ ] Pharmacist
[ ] Physician
[ ] Physician Assistant
[ ] Dietitian or Nutritionist
[ ] Mental/Behavioral Health Professional
[ ] Substance Abuse Professional
[ ] Social Worker or Case Manager
[ ] Community Health Worker (includes Peer Educator or Navigator)
[ ] Clergy or Faith-based Professional
[ ] Practice Administrator or Leader (e.g., Chief Executive Officer, Nurse Administrator)
[ ] Other allied health professional (e.g., Medical Assistant, Podiatrist, Physical Therapist), please specify: ____
[ ] Other Public Health Professional, please specify: __________________________________
[ ] Other Non-Clinical Professional (e.g., Front Desk Staff, Grant Writer), please specify: ____

Skip to Question 18.

Thinking about your work setting and employment role, please answer the following questions:

12. What is the state/territory and zip code of your employment setting?

___ ___*(state/territory)* and ___ ___ ___ ___ ___*(zip code)*

13. Does your principal employment setting receive Ryan White HIV/AIDS Program funding?

[ ] Yes
[ ] No
[ ] Not sure

14. Are HIV prevention, care, or treatment services provided by your principal employment setting?

[ ] Yes
[ ] No
[ ] Not sure

15. What is your principal employment setting? *(Select one)*

[ ] Clinic
[ ] Hospital
[ ] Pharmacy
[ ] Public Health Agency (government or other)
[ ] Other healthcare setting, please specify: ____
[ ] Not currently employed in a healthcare setting, please specify: ___________________________

16. What is your primary profession/occupation? *(Select one)*

[ ] Dentist
[ ] Other Dental Professional
[ ] Nurse Practitioner or other Nursing Professional who prescribes
[ ] Nurse Professional who does not prescribe
[ ] Midwife
[ ] Pharmacist
[ ] Physician
[ ] Physician Assistant
[ ] Dietitian or Nutritionist
[ ] Mental/Behavioral Health Professional
[ ] Substance Abuse Professional
[ ] Social Worker or Case Manager
[ ] Community Health Worker (includes peer educator or navigator)
[ ] Clergy or Faith-based Professional
[ ] Practice Administrator or Leader (e.g., Chief Executive Officer, Nurse Administrator)
[ ] Other allied health professional (e.g., Medical Assistant, Podiatrist, Physical Therapist), please specify: ____
[ ] Other Public Health Professional, please specify: ____
[ ] Other non-clinical professional (e.g., Front Desk Staff, Grant Writer), please specify: ____

17. In your work setting, do you have direct interaction with clients/patients?

[ ] Yes *(Continue to Question 17a)*
[ ] No *(SURVEY COMPLETE. THANK YOU!)*

17a. In your role at your work setting….

	Yes	No
Do you provide HIV prevention and testing services to clients/patients?	○	○
Do you screen for and prescribe HIV PrEP to clients/patients?	○	○
Do you provide services directly to people with HIV?	○	○

USE OF AETC PROGRAMS

18. **The AETC Program includes eight Regional AETCs, two national centers, and the National HIV Curriculum. In the *past 12 months*, have you used any resources and/or trainings from the following AETC Programs?**

	Yes	No	Unsure	Not Aware of Program
National Coordinating Resource Center (NCRC)	○	○	○	○
National Clinician Consultation Center (NCCC)	○	○	○	○
National HIV Curriculum (NHC) e-Learning Platform Programs	○	○	○	○

Thank you for completing this survey!

To Be Completed by AETC

AETC Region Number: ___ ___

Regional Partner Site Number: ___ ___ ___

Indicate Survey Phase:

____**Baseline**

____ **Follow-Up**

Date Form Completed (MM/DD/YYYY): ___ ___ / ___ ___ / ___ ___ ___ ___

Health Professional Program ID: ___ ___ ___

Please ensure the 3-digit HPP ID matches the 3-digit HPP ID on the ER to support data linkage.
