# Supplementary material for: Interprofessional Education: An Innovative Approach to Increase the Human Immunodeficiency Virus Workforce
Source: Open Forum Infect Dis. 2023 Nov 7;10(11):ofad560. doi: 10.1093/ofid/ofad560 (PMC10665034; doi:10.1093/ofid/ofad560)
Supplement: ofad560_Supplementary_Data [file ofad560_supplementary_data.zip › Supplementary Material 1 (Guidelines and Learning Objectives of the SE AETC IPE Program).docx]

**Supplementary Material 1: Guidelines and Learning Objectives of the SE AETC IPE Program**

The following guidelines and learning objectives were developed by the SE AETC IPE program, and based on four broad Interprofessional Education Collaborative (IPEC) Competencies.

*Guidelines*

1. Cultivate respectful professionals
2. Prepare a collaborative-practice-ready work force
3. Improve health care delivery and systems
4. Create self-directed lifelong learners

*Learning Objectives*

1. Describe the historical context of HIV infection globally and in the US.
2. Recall the basics regarding HIV pathogenesis.
3. Discuss the epidemiology of HIV infection and how it may differ in the US from the rest of the world.
4. Identify who should be tested for HIV infection.
5. Explain indications for treatment of HIV.
6. List barriers to effective HIV diagnosis and treatment (i.e., the HIV care continuum).
7. Identify the social determinants of health that can uniquely impact people living with HIV
8. Utilize available health resources in a responsible manner to provide quality care and support services for PLWH
9. Articulate how interprofessional teams of health students can best help people living with HIV
